# Auranofin Attenuates Non-Alcoholic Fatty Liver Disease by Suppressing Lipid Accumulation and NLRP3 Inflammasome-Mediated Hepatic Inflammation In Vivo and In Vitro

**DOI:** 10.3390/antiox9111040

**Published:** 2020-10-23

**Authors:** Hyun Hwangbo, Min Yeong Kim, Seon Yeong Ji, So Young Kim, Hyesook Lee, Gi-Young Kim, Cheol Park, Young-Sam Keum, Su Hyun Hong, Jaehun Cheong, Yung Hyun Choi

**Affiliations:** 1 Department of Molecular Biology, Pusan National University, Busan 46241, Korea; hbhyun2003@naver.com (H.H.); 14731@deu.ac.kr (S.Y.K.); 2 Department of Biochemistry, College of Korean Medicine, Dong-eui University, Busan 47227, Korea; hongsh@deu.ac.kr; 3 Anti-Aging Research Center, Dong-eui University, Busan 47340, Korea; ilytoo365@deu.ac.kr (M.Y.K.); 14602@deu.ac.kr (S.Y.J.); 14769@deu.ac.kr (H.L.); 4 Department of Marine Life Sciences, School of Marine Biomedical Sciences, Jeju National University, Jeju 63243, Korea; immunkim@jejunu.ac.kr; 5 Division of Basic Sciences, College of Liberal studies, Dong-eui University, Busan 47340, Korea; parkch@deu.ac.kr; 6 College of Pharmacy and Integrated Research Institute for Drug Development, Dongguk University, Goyang 10326, Korea; keum03@dongguk.edu

**Keywords:** auranofin, NAFLD, hepatic inflammation, lipid accumulation, NLRP3 inflammasome

## Abstract

Non-alcoholic fatty liver disease (NAFLD) causes liver dysfunction and is associated with obesity and type 2 diabetes. Chronic inflammation is associated not only with the development of NAFLD, but also with hepatic diseases, including steatohepatitis, cirrhosis, and hepatocellular carcinoma. Auranofin is a treatment for rheumatoid arthritis and has recently been reported to have potential effects against a variety of diseases, including inflammation, cancer, and viral infection. In this study, auranofin may be considered as a new treatment for the management of metabolic syndrome, as well as in the treatment of NAFLD through immunomodulation. To determine the effect of auranofin on NAFLD, C57BL/6 mice were randomly grouped, fed a regular diet or a high fat diet (HFD), and injected with normal saline or auranofin for 8 weeks. Auranofin significantly decreased the body weight, epididymal fat weight, serum aspartate aminotransferase (AST), and glucose, as well as the serum triglyceride, cholesterol, and low-density lipoprotein cholesterol levels as compared to the HFD group. We also observed that hepatic steatosis was increased in the HFD group and was suppressed by auranofin treatment. In addition, auranofin suppressed the expressions of interleukin (IL)-1β, IL-18, caspase-1, and the NOD-like receptor family pyrin domain containing 3 (NLRP3) in the liver tissue. Furthermore, the expression of NADPH oxidase 4 and peroxisome proliferator-activated receptor γ (PPARγ), which are a major source of oxidative stress and a regulator of adipogenesis, respectively, were also decreased by auranofin. In addition, primary mouse hepatocytes were incubated with lipopolysaccharide (LPS) and palmitic acid (PA) to induce lipid accumulation and hepatic inflammation for an in vitro model. Auranofin could significantly inhibit LPS- and PA-induced inflammatory activity including nitric oxide and NLRP3 inflammasome-mediated cytokines. The results of this study demonstrate that auranofin treatment inhibits the characteristics of NAFLD through the inhibition of NLRP3 inflammasome. Therefore, auranofin may have potential as a candidate for improving NAFLD symptoms.

## 1. Introduction

Non-alcoholic fatty liver disease (NAFLD) is a metabolic disease caused by the excessive storage of fat in the liver, not liver disease caused by other causes such as alcohol, drugs and virus [1,2]. It is also reported that NAFLD increases exacerbated chronic inflammation and oxidative stress, causing dysfunction in liver tissues. There is increasing evidence that it is actually a multi-system disease that affects organ and control pathways between multiple systems [3,4,5,6]. The NAFLD process develops into nonalcoholic steatohepatitis (NASH) by lipid accumulation in hepatocytes, which causes reactive oxygen species, lipid peroxidation, and proinflammatory cytokines leading to hepatic injury and inflammation [7,8]. Rarely, NAFLD can advance into steatohepatitis, fibrosis and cirrhosis, and hepatocellular carcinoma [9,10]. Therefore, NAFLD, which has the ability to develop from metabolic syndrome to steatohepatitis and hepatocellular carcinoma, requires an effective treatment [11,12]. Changes in diet or exercise may delay the progression of NAFLD but taking medication can also be effective [13,14]. However, no drug has been designed that prevents and treats NAFLD effectively. Thus, it is important to understand how NAFLD progresses so we can develop an effective treatment.

The NOD-like receptor family pyrin domain containing 3 (NLRP3) belongs to the NOD-like receptor (NLR) subfamily of pattern recognition receptors and constitutes a multicomplex as an NLRP3 inflammasome with ASC (the adaptor molecule apoptosis-associated speck-like protein containing a CARD) and pro-caspase-1 [15,16]. The NLRP3 inflammasomes are sensors for various pathogen-associated molecular patterns and damage-associated molecular patterns and produce the inflammatory cytokines including interleukin (IL)-1β and IL-18 by activating caspase-1 [16,17,18]. Several studies have demonstrated that the NLRP3 inflammasome induces chronic inflammation in metabolic diseases, and it is also associated with the progression of NALFD and NASH [19,20,21].

Gold-based compounds have been used in a wide range of treatments for a long time, and among them, their role as a treatment for rheumatoid arthritis is the most representative [22,23]. These compounds are suggested for their immunosuppressive activity by inhibiting immune cell activation [24,25]. Auranofin is a gold-containing compound and an FDA-approved drug for the treatment of rheumatoid arthritis and has been studied for potential preventive applications in several other diseases, including cancer, neurodegenerative disorders, pathogenic infections, and inflammation [26,27,28]. Auranofin can also inhibit phagocytosis by macrophages and can reduce proinflammatory cytokines through the inhibition of nuclear factor-kappa B activation [29,30,31]. In addition, auranofin has the antioxidant property of enhancing the activation of nuclear factor erythroid-2-related factor 2 [32,33,34]. However, to date, no research has been done on whether auranofin can improve NAFLD. Therefore, the purpose of this study is to determine the effect of auranofin on hepatic steatosis, inflammation and NLRP3 inflammasome, which contribute to the development of NALFD in both in vivo and in vitro.

## 2. Materials and Methods

### 2.1. Materials

Auranofin, protease inhibitor cocktail, collagenase, lipopolysaccharide (LPS), and palmitic acid (PA) were purchased from Sigma-Aldrich Chemical Co. (St. Louis, MO, USA). Formaldehyde was purchased from Junsei Chemical Co., Ltd. (Tokyo, Japan). Paraffin was purchased from Leica Biosystems Co. (Wetzlar, Germany). Xylene, dithiothreitol, Tween 20, and dimethyl sulfoxide (DMSO) were purchased from Amresco Inc. (Solon, OH, USA). VECTASTAIN ABC kits and 3, 3-diaminobenzidine (DAB) were purchased from Vector Laboratories Inc. (Burlingame, CA, USA). Nitrocellulose membrane was purchased from GE Healthcare (Illinois, IL, USA). Skim milk and anti-inducible nitric oxide synthase (iNOS) were purchased from BD Biosciences (Franklin Lakes, NJ, USA). Antibodies against F4/80, IL-1β, IL-18, NLRP3 and NADPH oxidase 4 (NOX4) were purchased from Abcam (Cambridge, U.K.). Anti-β-actin antibody was purchased from Bioworld Technology, Inc. (Nanjing, China). Anit-caspase-1 and peroxisome proliferator-activated receptor γ (PPARγ), and secondary antibodies were purchased from Santa Cruz Biotechnology, Inc. (Dallas, TX, USA). Electrochemiluminescence (ECL) reagent and primary hepatocyte maintenance supplements were purchased from Thermo Fisher Scientific (Waltham, MA, USA). IL-1β, IL-18, tumor necrosis factor (TNF)-α, and IL-6 enzyme-linked immunosorbent assay (ELISA) kits were purchased from R&D Systems (Minneapolis, MN, USA). Williams E medium and no phenol red were purchased from GIBCO BRL (Grand Island, NY, USA). Six-well and 96-well plates were purchased from SPL Inc. (Houston, TX, USA). Finally, 3-(4,5-dimethylthiazol-2-yl)-2,5-diphenyltetrazolium bromide (MTT) was purchased from Invitrogen (Waltham, MA, USA). All other chemicals were purchased from Sigma-Aldrich Chemical Co.

### 2.2. Animal Models

Male C57BL/6 mice (6 weeks old) were purchased from Samtako Bio Korea (Osan, Republic of Korea) with an initial body weight of 20 g. The animals were maintained at a temperature of 22 ± 2 °C and a humidity of 70 ± 5% under a regular 12 h light/dark cycle. Animal experiments were conducted in accordance with the Animal Experimental Ethics Committee of Dong-eui University (Confirmation number: R2017-005). Mice were allowed to acclimate for a week with free access to water and a standard mouse chow. They were randomly divided into four groups (*n* = 10) as follows: a regular diet + intraperitoneal injections (IP) of normal saline (JW Pharmaceutical, Seoul, Korea, normal group), a high-fat diet (containing 60% kcal fat, HFD) + IP of normal saline (HFD group), HFD + IP of auranofin (HFD + AF group), and a regular diet + IP of auranofin (AF group). The mice of the normal group were given a regular diet (#D12450K) and the mice of all HFD treatment groups consumed HFD (#D12492) containing 60% kcal fat. The experimental diets were purchased from Research Diets (New Brunswick, NJ, USA). The mice had an intake and daily intraperitoneal injection of normal saline and auranofin (10 mg/kg) for 8 weeks [35]. All experimental animals were acclimatized for a week, then the HFD fed and auranofin injection were started at the same time. The body weight was monitored once a week during the experimental period. At the end of the experiments, all animals were sacrificed using CO_2_, and the harvested livers were stored at −80 °C and fixed with 4% formaldehyde until further analysis.

### 2.3. Biochemical Analysis

For serum biochemical analysis, whole blood was collected from the heart. Among the 10 samples per group, two samples were dropped out of the blood collection process, the eight samples were used for further analysis. Serum was collected by centrifugation of blood samples at 3000 rpm for 10 min at 4 °C and stored at −80 °C for subsequent analysis. Serum liver function levels, including aspartate aminotransferase (AST) and blood fat including glucose, triglyceride, total lipid, total cholesterol, and low-density lipoprotein (LDL) cholesterol were examined at the Green Cross Lab. (Yongin, Republic of Korea). AST, glucose, and LDL cholesterol were analyzed using the Fuji dri-chem slide kit (Fuji Film Co., Tokyo, Japan) with Dri-chem 3500s (Fuji Film Co.), an automatic blood biochemical test analyzer, and triglycerides were measured using a triglycerides reagents kit (Bayer Healthcare LLC, Los Angeles, CA, USA). Total cholesterol was analyzed with a cholesterol reagent kit (Bayer AG Co., Leverkusen, Germany) using ADVIA 1650 biochemistry analyzer (Bayer AG Co.).

### 2.4. Histology and Immunohistochemistry

The liver tissues were fixed in 4% formaldehyde for two days and embedded in paraffin. The section of blocks was cut into thickness of 4 μm using a microtome (Leica RM2125, Leica Biosystems, Heidelberg, Germany). For hematoxylin and eosin (H&E) staining, the sections were deparaffinized rehydrated, and washed with distilled water to allow the hydrophilic solution to penetrate. After tissue watering, H&E staining was performed to observe liver tissue histological changes. For immunohistochemistry analysis, the sections were deparaffinized, rehydrated, cooked in antigen retrieval solution (Abcam), and dipped in 3% hydrogen peroxide solution for 30 min. Specific primary antibodies were then applied for 1 h at room temperature, and the sections were incubated with secondary antibodies for 40 min. Immunoreactions were visualized with DAB staining, and the sections were counterstained with Mayer’s hematoxylin. The stained sections were dehydrated, mounted and observed using the EVOS FL Auto 2 imaging system (Thermo Fisher Scientific). Densitometric analysis of the data was performed using the ImageJ^®^ software (v1.48, NIH, Bethesda, MD, USA).

### 2.5. Western Blot Analysis

The liver tissues were washed with cold phosphate-buffered saline (PBS) to remove blood and homogenized with a lysis buffer (250 mM NaCl, 25 mM Tris-Cl (pH 7.5), 5 mM ethylenediaminetetraacetic acid (pH 8.0), 1% NP-40, 1 mM 4-(2-aminoethyl) benzenesulfonyl fluoride hydrochloride, 5 mM of dithiothreitol, and protease inhibitor cocktail). Then the lysates were centrifuged at 14,000 rpm at 4 °C for 30 min. The supernatants were acquired, and the concentration of proteins was estimated with a protein assay dye reagent. The protein samples were quantified at the same concentration and stored at −20 °C until use. The quantified samples (40 μg) were separated with sodium dodecyl sulfate-polyacrylamide gel electrophoresis and transferred onto a nitrocellulose membrane. The membranes were blocked with 5% skim milk in PBS containing 0.1% Tween 20 (PBST) for 1 h and then probed with the appropriate concentrations of primary antibodies overnight at 4 °C. After washing three times with PBST, the membranes were reacted with secondary antibodies (anti-mouse or anti-rabbit) for 1 h at room temperature, and the proteins were visualized using ECL. Densitometric analysis of the data was performed using the ImageJ^®^ software.

### 2.6. Cytokines Assays

To measure the cytokine level, the liver tissues were homogenized and lysed, and culture supernatants from primary hepatocytes were collected and frozen at −80 °C before use. The cytokines including IL-1β, IL-18, TNF-α, and IL-6 were measured according to the ELISA kit manufacturer’s instructions. The absorbance was measured at a 450 nm wavelength using a microplate reader (Beckman Coulter Inc., Brea, CA, USA) at Core-Facility Center for Tissue Regeneration, Dong-eui University (Busan, Republic of Korea).

### 2.7. Isolation of Hepatocytes

C57BL/6 primary hepatocytes were isolated by perfusion through the hepatic portal vein, as previously described [36]. The liver was perfused with ethylene glycol-bis(2-aminoethylether)-N,N,N′,N′-tetra acetic acid (EGTA) buffer (5.4 mM KCl, 0.44 mM KH_2_PO_4_, 140 mM NaCl, 0.34 mM Na_2_HPO_4_, 0.5 mM EGTA, and 25 mM of Tricine) to eject blood and 0.075% collagenase to digest liver. All buffers were perfused at 5 mL/min for 10 min. The digested liver tissue was filtered and centrifugated three times at 750 rpm for 5 min. The cells were cultured in Williams E medium with no phenol red supplemented with primary hepatocyte maintenance supplements and incubated overnight at 37 °C in 5% CO_2_.

### 2.8. Chemical Treatment and Cell Viability Assay

The primary hepatocytes (5 × 10^5^ cells/well) were seeded in a collagen-coated six-well plate and pretreated with auranofin (0.5, 1, and 1.5 μM) for 1 h, and treated with 1 μg/mL of LPS and 250 μM of PA for 48 h. After exposure of chemicals, the MTT solution was diluted to 0.5 mg/mL and added to the medium, followed by reaction for 3 h. All of the supernatant was removed, the formazan crystal was dissolved in DMSO and the absorbance was measured at a wavelength of 540 nm using a microplate reader (Molecular Device Co.) as previously described [36].

### 2.9. Nitrite Production Measurement

The nitrite, a stable metabolite of nitric oxide (NO), was measured to detect NO production. The supernatant of LPS and PA-induced primary hepatocytes culture was mixed with an equal volume of Griess reagent (0.5% sulfanilamide and 0.05% N-1-naphthylethylenediamine) and incubated in a 96-well plate for 10 min at room temperature. The absorbance was determined at 540 nm, using a microplate reader (Molecular Device Co.). The nitrite concentration was calculated with a standard curve, using sodium nitrite (NaNO_2_) [37].

### 2.10. Statistical Analysis

Statistical analysis was performed with the GraphPad Prism 5.03 (GraphPad Software, Inc., San Diego, CA, USA) using one-way analysis of variance (ANOVA) for multiple comparisons, followed by Tukey’s post hoc test. All numerical data are presented as mean ± standard deviation (SD) in triplicate experiments. Values of *p* < 0.05 were considered to be statistically significant.

## 3. Results

### 3.1. Auranofin Prevents Body Weight and Epididymal Fat Pad in HFD-Induced NAFLD Model

**Figure 1:**
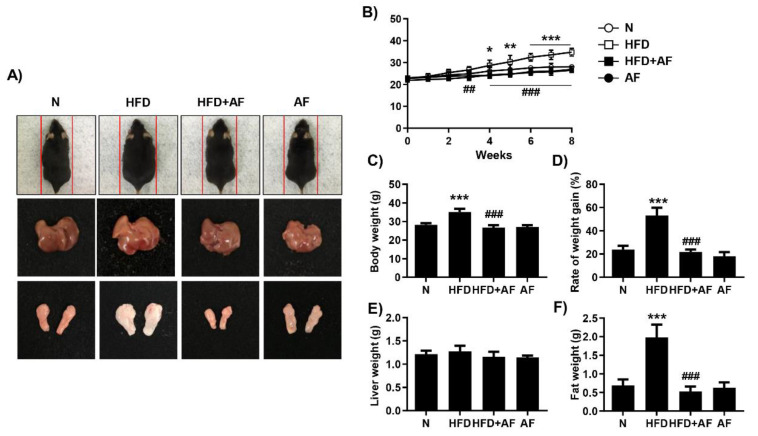
Auranofin attenuates body weight gain and the epididymal fat pad in the high-fat diet (HFD)-induced non-alcoholic fatty liver disease (NAFLD) mouse model. Animals were fed a 60% HFD (60% of calories from fat) and/or a regular diet and also received intraperitoneal injection of auranofin (10 mg/kg) and/or normal saline for 8 weeks. (**A**) Representative photographic images of the whole mouse body, liver, and epididymal fat pad specimens in each group. (**B**) Body weight gain for 8 weeks. Data are expressed as the mean ± SD (*n* = 10). * *p* < 0.05, ** *p* < 0.01, and *** *p* < 0.001 compared with the normal group. ^##^ *p* < 0.01 and ^###^ *p* < 0.001 compared with HFD group. (**C**–**F**) The average values for body weight, rate of weight gain, liver and epididymal fat pad after the study period. Data are expressed as the mean ± SD (*n* = 8). *** *p* < 0.001 compared with the normal group. ^###^ *p* < 0.001 compared with HFD group. N, normal group; HFD, HFD fed group; HFD+AF, HFD fed and auranofin injection group; AF, auranofin injection group.

To confirm the improvement effect of auranofin on NALFD, the results were obtained at the end of the experiment after administration of mice with or without HFD and with auranofin or normal saline for 8 weeks. The whole-body size and weight of the HFD group increased as compared to the control group (Figure 1A–C). However, treatment with auranofin significantly suppressed HFD-induced weight gain (Figure 1D). In addition, liver weight did not increase significantly, although it showed a tendency to increase, and the size and weight of the epididymal fat pad were dramatically decreased in the auranofin + HFD group as compared to HFD group (Figure 1A,E,F).

### 3.2. Auranofin Reduces Lipid Accumulation in Serum and Hepatic Steatosis in the HFD-Induced NAFLD Model

**Figure 2:**
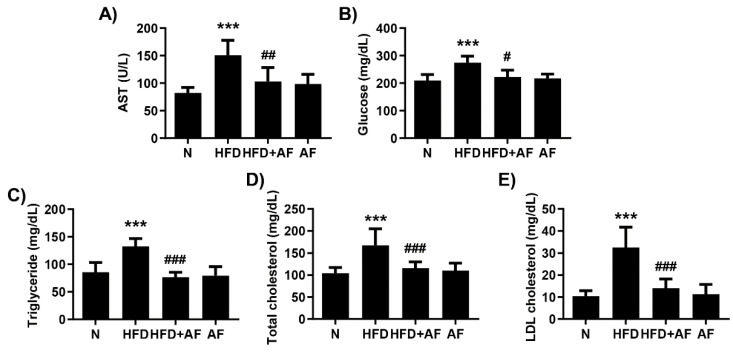
Auranofin improves HFD-induced suppression of the hepatic function and lipid accumulation in serum. Following treatment with or without HFD and auranofin for 8 weeks, the blood was collected, and the serum was analyzed. (**A**–**E**) The serum analysis results are expressed in the graph. Data are expressed as the mean ± SD (*n* = 8). *** *p* < 0.001 compared with the normal group. ^#^ *p* < 0.05, ^##^ *p* < 0.01, and ^###^ *p* < 0.001 compared with HFD group. N, normal group; HFD, HFD fed group; HFD+AF, HFD fed and auranofin injection group; AF, auranofin injection group.

**Figure 3:**
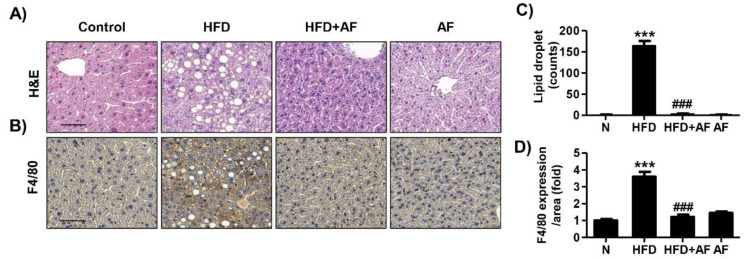
Auranofin alters morphological changes and macrophage infiltration in the liver of the HFD-induced NALFD mouse model. (**A**) The histological changes were assessed by H&E staining. Scale bar—50 μm. (**B**) The expressions of F4/80 were evaluated by immunohistochemistry (IHC) using specific antibodies. Representative images of F4/80-positive cells. Scale bar—50 μm. (**C**) The lipid droplets per area were counted and expressed in graph. (**D**) Quantification of the F4/80 expression in area. Data are expressed as means ± SD (*n* = 3). *** *p* < 0.001 compared with the normal group. ^###^ *p* < 0.001 compared with HFD group. N, normal group; HFD, HFD fed group; HFD+AF, HFD fed and auranofin injection group; AF, auranofin injection group.

Serum biochemical analysis was examined to investigate the liver function and the serum lipid profile. The AST level, a parameter of liver function, increased in HFD group but was significantly decreased by auranofin (Figure 2A). The administration of auranofin also led to a decrease in the serum glucose, triglyceride, total cholesterol, and LDL cholesterol as compared to the HFD group (Figure 2B–E). Furthermore, liver tissue with H&E staining (Figure 3A) and F4/80 immunostaining (Figure 3B) decreased lipid droplet accumulation and macrophage infiltration in the auranofin + HFD group as compared to HFD group. Therefore, these results showed that auranofin prevents serum lipid accumulation, hepatic steatosis, and macrophage infiltration, which were increased by HFD.

### 3.3. Auranofin Decreases HFD-Induced IL-1β and IL-18 Expression in the NAFLD Model

**Figure 4:**
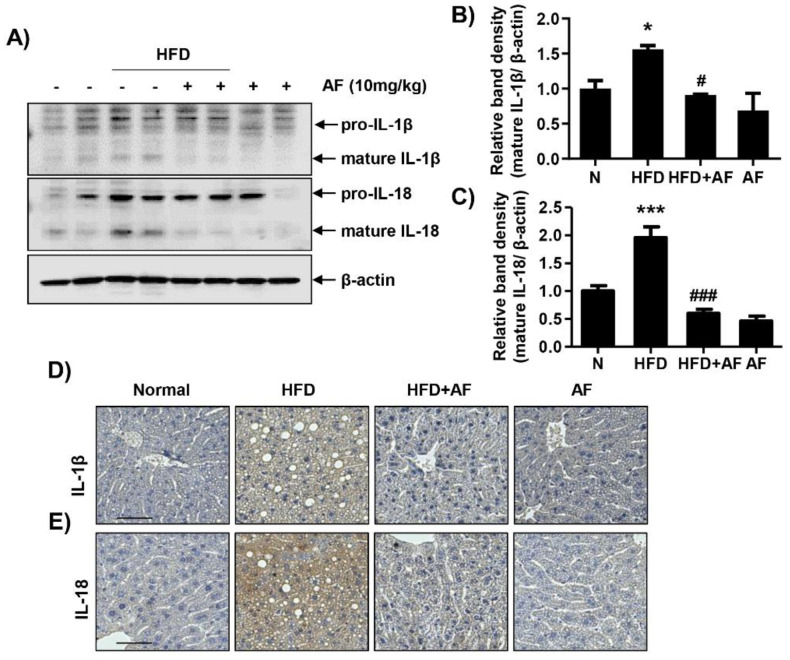
Auranofin ameliorates interleukin (IL) 1-β and IL-18 expression and secretion in the liver and serum of the HFD-induced NAFLD mouse model. Following treatment with or without HFD and auranofin for 8 weeks, the liver tissues were obtained. (**A**) Liver (300 μg) was homogenized with 500 μL of lysis buffer supplemented with protease and phosphatase inhibitors. Western blots showing levels of IL-1β and IL-18 in the mouse liver in each group. Quantification of IL-1β (**B**) and IL-18 (**C**) expression. Data are expressed as means ± SD (*n* = 4). * *p* < 0.05 and *** *p* < 0.001 compared with the normal group. ^#^ *p* < 0.05 and ^###^ *p* < 0.001 compared with HFD group. The hepatic expression of IL-1β (**D**) and IL-18 (**E**) protein were assessed by immunohistochemistry analysis (*n* = 3). Scale bar—50 μm. N, normal group; HFD, HFD fed group; HFD+AF, HFD fed and auranofin injection group; AF, auranofin injection group.

The expressions of cytokines were investigated since an HFD had increased inflammatory cell infiltration. IL-1β and IL-18 expressions, which are representative of proinflammatory cytokines and are secreted upon NLRP3 inflammasome formation, were confirmed by Western blot and immunostaining. The expressions of IL-1β and IL-18 were significantly decreased in auranofin + HFD group compared to HFD group (Figure 4). Therefore, auranofin reduced the HFD-induced expressions of cytokines and could suggest a correlation with NLRP3 inflammasome.

### 3.4. Auranofin Suppresses HFD-Induced NLRP3 Inflammasome, NOX4 and PPARγ in the NAFLD Model

**Figure 5:**
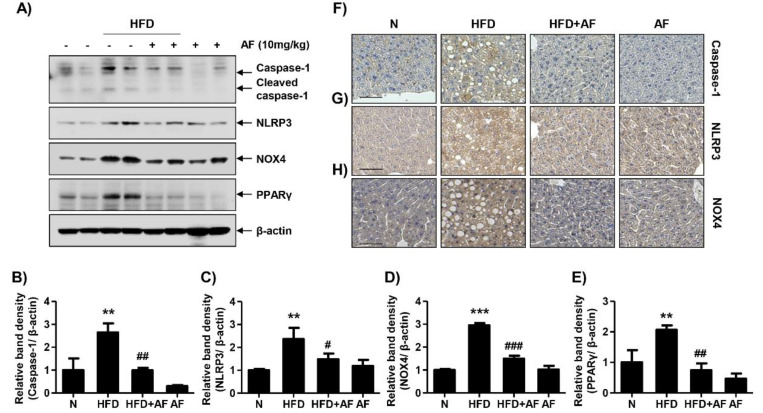
Auranofin reduces NOD-like receptor family pyrin domain containing 3 (NLRP3) inflammasome-associated proteins, NADPH oxidase 4 (NOX4) and peroxisome proliferator-activated receptor γ (PPARγ) expression in the liver of the HFD-induced NAFLD mouse model. (**A**) Western blots of caspase-1, NLRP3, NOX4 and PPARγ were performed with the liver tissue. (**B**–**E**) Quantification of activated caspase-1, NLRP3, NOX-4 and PPARγ. Data are expressed as means ± SD (*n* = 4). ** *p* < 0.01 and *** *p* < 0.001 compared with the normal group. ^#^ *p* < 0.05, ^##^ *p* < 0.01, and ^###^ *p* < 0.001 compared with HFD group. (**F**–**H**) To evaluate the hepatic expression of caspase-1, NLRP3 and NOX4, paraffin blocks of liver were section into 4 μm thicknesses and stained with specific antibodies (*n* = 3). Scale bar—50 μm. N, normal group; HFD, HFD fed group; HFD+AF, HFD fed and auranofin injection group; AF, auranofin injection group.

The association between HFD-induced NAFLD and NLRP3 inflammasome was investigated by Western blot and IHC staining. In addition, the expression of NOX4, which induces oxidative stress and forms the NLRP3 inflammasome, was examined. Additionally, PPARγ, which stimulates adipogenesis, were also confirmed. As shown in Figure 5, the expressions of active caspase-1 and NLRP3, which form the NLRP3 inflammasome complex, were increased in HFD-group. Notably, the expressions of NOX4 and PPARγ were increased in the HFD group. However, HFD-induced proteins, including caspase-1, NLRP3, NOX4 and PPARγ, were decreased by the auranofin treatment. These results indicate that auranofin inhibited HFD-induced lipid metabolism and inflammation-related proteins by mediating NLRP3 inflammasome.

### 3.5. LPS and PA Stimulate Inflammatory Cytokines in Primary Hepatocytes

**Figure 6:**
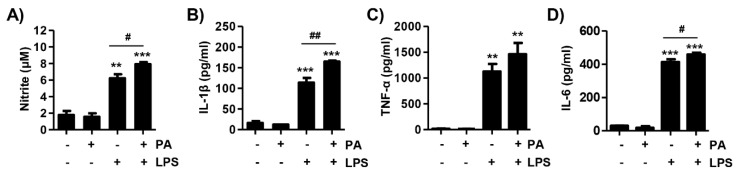
Lipopolysaccharide (LPS) and palmitic acid (PA) induce nitrite release and proinflammatory cytokines in primary hepatocytes. The cells were isolated from C57BL/6 mice and treated with LPS (1 μg/mL) and PA (250 μM) for 24 h. Following incubation, the cell supernatant was collected and used for analysis. (**A**) The release of nitrite was measured by Griess reagent. The absorbance was read using a microplate reader at 540 nm. Data are expressed as means ± SD (*n* = 6). (**B**–**D**) The level of IL-1β, tumor necrosis factor (TNF)-α and IL-6 secretions was evaluated with an ELISA kit. The absorbance was read using the microplate reader at 450 nm and the optical density was calculated as described by the manufacturer’s protocols. The data are the average of three independent experiments. The results are represented by a mean ± SD (*n* = 6). Statistical analysis was performed using one-way analysis of variance (ANOVA) with Tukey’s post-hoc test. ** *p* < 0.01 and *** *p* < 0.001 compared with un-treated cells. ^#^ *p* < 0.05 and ^##^ *p* < 0.01 compared with LPS-treated cells. PA, palmitic acid; LPS, lipopolysaccharide.

The secretion of cytokines plays an important role in hepatic inflammation. Thus, this study was conducted to confirmed whether auranofin could inhibit LPS- and PA-induced hepatic inflammation using primary hepatocytes. As shown in Figure 6, LPS and PA together effectively induced the release of nitrite as compared to LPS or PA alone. Next, production of inflammatory cytokines was measured, and, as a result, LPS and PA increased the secretion of IL-1β, TNF-α, and IL-6. These results indicate that LPS and PA can induce secretion of inflammatory mediators in primary hepatocytes.

### 3.6. Auranofin Inhibits LPS and PA-Induced Inflammation in Primary Hepatocytes

**Figure 7:**
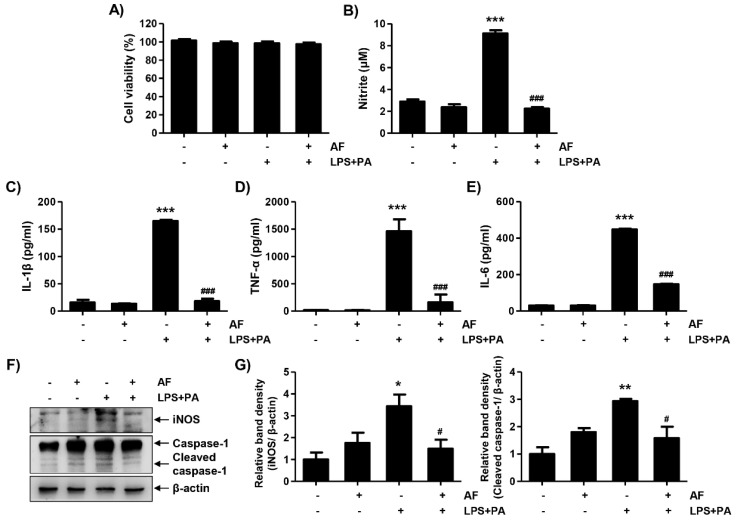
Auranofin suppresses LPS and PA-induced inflammation in primary hepatocytes. The cells were pretreated with auranofin (1.5 μM) for 1 h and treated with LPS and PA for 24 h. (**A**) Cell viability was estimated by 3-(4,5-dimethylthiazol-2-yl)-2,5-diphenyltetrazolium bromide (MTT) assay. The absorbance was measured using the microplate reader at 540 nm and compared to the control which was set to 100%. Data are expressed as means ± SD (*n* = 6). (**B**–**E**) Nitrite and proinflammatory cytokines, including IL-1β, TNF-α and IL-6, were assessed using Griess reagent and ELISA kit. The absorbance was measured using the microplate reader. The error bars represent the standard deviation of three independent experiments. Statistical analysis was performed using an ANOVA with Tukey’s post-hoc test (*n* = 6). (**F**) The cells were lysed, and a Western blot was performed to evaluate the expression of inducible nitric oxide synthase and caspase-1. Equal protein loading was confirmed by β-actin expression. (**G**) Quantification of iNOS and activated caspase-1 expression. Data are expressed as means ± SD (*n* = 2). * *p* < 0.05, ** *p* < 0.01, and *** *p* < 0.001 compared with un-treated cells. ^#^ *p* < 0.05 and ^###^ *p* < 0.001 compared with LPS and PA-treated cells. AF, auranofin; PA, palmitic acid; LPS, lipopolysaccharide.

To investigate the inhibitory effects of auranofin on inflammatory response, cytokine levels were confirmed using LPS- and PA-stimulated primary hepatocytes. LPS and PA promoted the secretion of nitrite and cytokines including IL-1β, TNF-α, and IL-6 into the cell supernatant, but auranofin inhibited the release of nitrite and cytokines (Figure 7B–E). Auranofin also decreased the expression of iNOS and active caspase-1 (Figure 7F,G). Therefore, these results demonstrate that auranofin inhibits the LPS- and PA-induced hepatic inflammation in the primary hepatocytes.

## 4. Discussion

NAFLD initiates with simple steatosis and can be the most common cause of chronic liver diseases such as steatohepatitis and fibrosis [38,39]. Many studies have reported that lipotoxicity and inflammation caused by continued lipid accumulation have an influence on the development process of NAFLD [40,41,42]. However, there is no effective treatment for NAFLD yet. Therefore, this study demonstrates that HFD-induced NAFLD was improved by suppressing hepatic inflammation by using auranofin, which has an anti-inflammatory effect.

C57BL/6 mice were fed with an HFD, leading to the pathological hallmarks of NAFLD, including weight gain, increased fat in serum and lipid accumulation in hepatocytes [43,44]. The HFD group also tended toward obesity because of the HFD, resulting in a significant increase in weight gain and the epididymal fat pad. In all groups, there was no change in the weight of the liver following 8 weeks of HFD intake, but the results of H&E staining did change, confirming the pathological liver tissue changes. Lipids were accumulated in the hepatocytes. However, there was a sufficient period for auranofin to improve changes in body weight, serum lipid concentration, and liver pathology. Furthermore, in the HFD group, recruitment of inflammatory cells was observed using the macrophage marker F4/80 antibody [45]. As many studies have reported, an increase in immune cells in NAFLD is a major cause of metabolic disease [46,47]. In this study, the inflammatory cell population was increased because of the HFD, but decreased by auranofin treatment. Thus, these results suggested that auranofin not only reduces the superficial lesions of NAFLD but also modulates its inflammatory components.

Accumulated evidence demonstrates that NLRP3 inflammasome was activated in HFD-induced NAFLD [48,49]. In general, NLRP3 inflammasome is composed of NLRP3, ASC, and pro-caspase-1, which activates caspase-1 [16,50]. Accordingly, activated caspase-1 could be secreted by converting pro-IL-1β and pro-IL-18 into bioactive cytokines [51]. In this study, auranofin, which has anti-inflammatory properties, suppressed the expression of IL-1 β and IL-18, the products of NLRP3 inflammasome in the HFD-induced NALFD model. Auranofin treatment also decreased the HFD-induced expression of caspase-1 and NLRP3. These results suggested that auranofin could regulate NLRP3 inflammasome formation in NAFLD induced by HFD. Moreover, auranofin reduced expression of NOX4, which is a catalytic enzyme that could generate H_2_O_2_ and mediates activation of NLRP3 inflammasome. Many studies also reported that NOX4 mediated in NLRP3 inflammasome formation [52,53]. HFD increased the expression of NLRP3 inflammasome and NOX4, and this mechanism was reduced by auranofin. Previous studies have shown that auranofin has antioxidant properties, and similarly, the results of this study also proved it inhibits NOX4, which causes oxidative stress [54]. However, further research is needed on this. Therefore, our results will add additional evidence to elucidate the association between NOX4 and NLRP3. In addition, auranofin significantly reduced the expression of PPARγ in HFD-induced NAFLD model. Thus, this study demonstrated that auranofin decreased hepatic steatosis by inhibiting PPARγ, which plays a key role in lipid storage and synthesis [55,56].

The isolated primary hepatocytes, treated with LPS and PA, were utilized to investigate the effect of auranofin on an in vitro model. Treatment with LPS and PA increased nitrite and secretions of proinflammatory cytokines, such as IL-1β, TNF-α, and IL-6, in primary hepatocytes. Similar to the results of the animal experiments, auranofin inhibits an inflammatory response, including the release of cytokines induced by LPS and PA in primary hepatocytes. Additionally, the expression of iNOS, an enzyme that produces nitrite oxide, and caspase-1, a protein related to NLRP3 inflammation, were also reduced. These results demonstrate that auranofin could work against inflammation induced by LPS and PA, and the association with NLRP3 inflammasome could also be demonstrated.

## 5. Conclusions

In conclusion, although further studies are necessary to evaluate the efficacy of auranofin at low doses and to identify the correlation with the result of animal study and clinical application, this study demonstrated that auranofin improves hepatic steatosis and inflammation in vivo and in vitro models. Therefore, this study suggests that auranofin can potentially prevent NAFLD by preventing the progression of NAFLD via NOX4-mediated NLRP3 inflammasome.
